# A Rare Case of Oropharyngeal Leech Infestation Causing Epistaxis and Dysphagia in a Child in Herat, Afghanistan: A Case Report

**DOI:** 10.1002/ccr3.73402

**Published:** 2026-08-30

**Authors:** Seyed Abdul Ahad Wadoodi, Said Abdul Kabir Hawari, Mohammad Masudi, Ali Rahimi, Nasar Ahmad Shayan

**Affiliations:** ^1^ Department of Internal Medicine, Faculty of Medicine Herat University Herat Afghanistan; ^2^ Department of Pediatrics, Faculty of Medicine Herat University Herat Afghanistan; ^3^ Department of Curative Medicine, Faculty of Medicine Jami University Herat Afghanistan; ^4^ Department of Public Health and Infectious Diseases, Faculty of Medicine Herat University Herat Afghanistan

**Keywords:** dysphagia, epistaxis, hirudiniasis, oropharyngeal leech infestation, untreated freshwater exposure

## Abstract

Oropharyngeal leech infestation is rare. A 12‐year‐old Afghan girl developed recurrent epistaxis, dysphagia, throat irritation, and cough after drinking untreated stream water. Examination revealed an 8‐cm leech attached to the posterior pharynx. Forceps removal immediately resolved symptoms. Leech infestation should be considered following freshwater exposure in endemic rural settings worldwide.

## Key Clinical Message

Clinicians should consider oropharyngeal leech infestation in children presenting with unexplained recurrent epistaxis, dysphagia, or throat symptoms after untreated freshwater exposure. Careful examination and prompt removal are essential because anticoagulant effects and possible airway extension can cause persistent bleeding, clinically important anemia, and potentially life‐threatening obstruction.

## 1 Introduction

Leech infestation of the upper aerodigestive tract, also known as hirudiniasis, is a rare but clinically important parasitic condition that is usually associated with drinking or swimming in untreated freshwater sources. Aquatic leeches may enter the body through contaminated water and attach to mucosal surfaces of the nose, nasopharynx, oropharynx, hypopharynx, larynx, trachea, or esophagus, where they feed on blood and produce local irritation, bleeding, and obstructive symptoms [1, 2, 3]. Although uncommon, reported cases show that leech infestation should be considered in patients from rural or underserved areas who present with unexplained epistaxis, hemoptysis, hematemesis, dysphagia, foreign‐body sensation, or respiratory distress [4, 5, 6].

Children are particularly vulnerable because they are more likely to drink unsafe water and because their smaller airway diameter increases the risk of obstruction and clinically significant blood loss. Pediatric reports have described leech infestation presenting with nasal bleeding, blood‐stained vomiting, anemia, and airway symptoms, sometimes after an initial misdiagnosis as pharyngitis, respiratory infection, or another common childhood condition [7, 8, 9]. Persistent bleeding occurs because leech saliva contains anticoagulant and vasodilatory substances, including hirudin, which may allow continued oozing even from a small attachment site [1, 2, 3]. Severe anemia requiring transfusion has been reported in infants and children, emphasizing the importance of early recognition and prompt removal [8, 10, 11].

In Afghanistan, unsafe water exposure remains possible in rural communities, yet published reports of oropharyngeal leech infestation are scarce. We present a rare case of oropharyngeal leech infestation causing epistaxis and dysphagia in a child from Herat, Afghanistan, to highlight this unusual but important diagnosis.

## 2 Case History/Examination

A 12‐year‐old girl with no known medical history presented to our clinic with a 1‐week history of recurrent epistaxis, progressive dysphagia, and intermittent coughing. Her symptoms began shortly after a picnic, during which she drank untreated stream water directly from the source while lying in a prone position. Soon afterward, she developed mild throat irritation, which gradually progressed to marked difficulty in swallowing and repeated episodes of nasal bleeding. Before attending our clinic, she had been evaluated by a local physician and treated symptomatically for a presumed nonspecific throat infection. However, her symptoms persisted and progressively worsened, prompting further clinical assessment 1 week after symptom onset.

**FIGURE 1:**
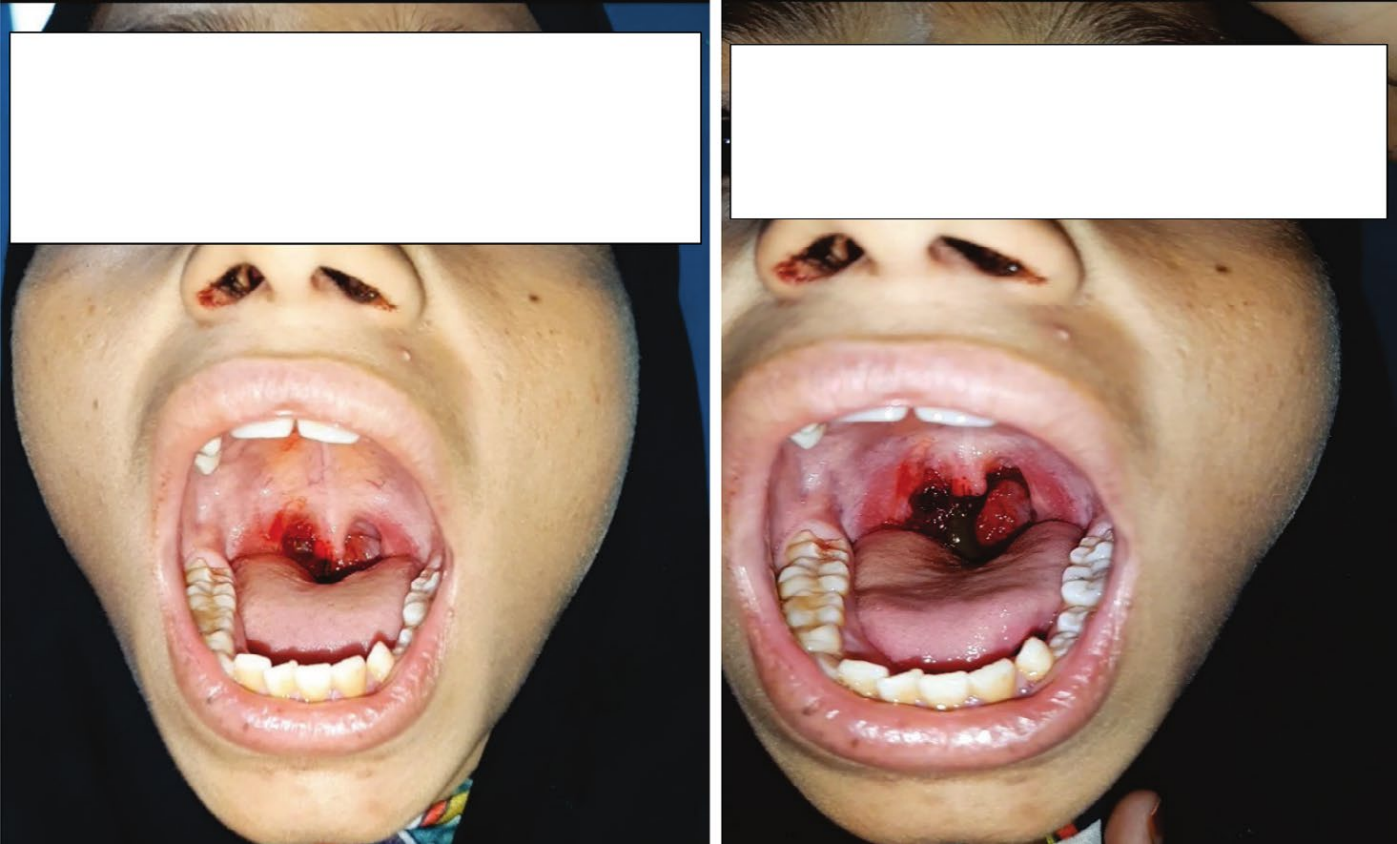
Oropharyngeal leech infestation before removal. Two clinical oropharyngeal views (A and B) show a dark‐colored live leech attached to the posterior pharyngeal mucosa and extending toward the hypopharynx in a 12‐year‐old girl with recurrent epistaxis and progressive dysphagia after drinking untreated stream water.

On presentation, the patient was hemodynamically stable and had no signs of respiratory distress. Initial oropharyngeal examination showed a small, dark‐colored structure adherent to the posterior pharyngeal wall. In view of the history of direct exposure to untreated freshwater and the unusual appearance of the lesion, a more meticulous examination was performed. This revealed a live leech firmly attached to the pharyngeal mucosa and extending from the oropharynx into the hypopharynx (Figure 1).

## 3 Methods (Differential Diagnosis, Investigations, and Treatment)

### 3.1 Differential Diagnosis

A nonspecific bacterial pharyngitis or tonsillitis had initially been presumed. At our assessment, the adherent dark structure and upper aerodigestive symptoms prompted consideration of a retained foreign body, adherent blood clot, necrotic tissue, peritonsillar lesion, vascular malformation, or parasitic infestation. Bacterial infection was less likely in the absence of typical inflammatory findings, and neither a foreign body nor a clot would be expected to move spontaneously. There was no unilateral peritonsillar swelling, uvular deviation, trismus, or muffled voice. The recent untreated freshwater exposure and direct visualization of a live, moving organism established the diagnosis of oropharyngeal leech infestation.

### 3.2 Investigations

Meticulous direct oropharyngeal examination demonstrated the attachment site and the extension of the leech toward the hypopharynx. Post‐removal laboratory evaluation showed a hemoglobin level of 9.7 g/dL, consistent with moderate anemia; the remaining complete blood count parameters and routine laboratory investigations were within normal limits.

### 3.3 Treatment

**FIGURE 2:**
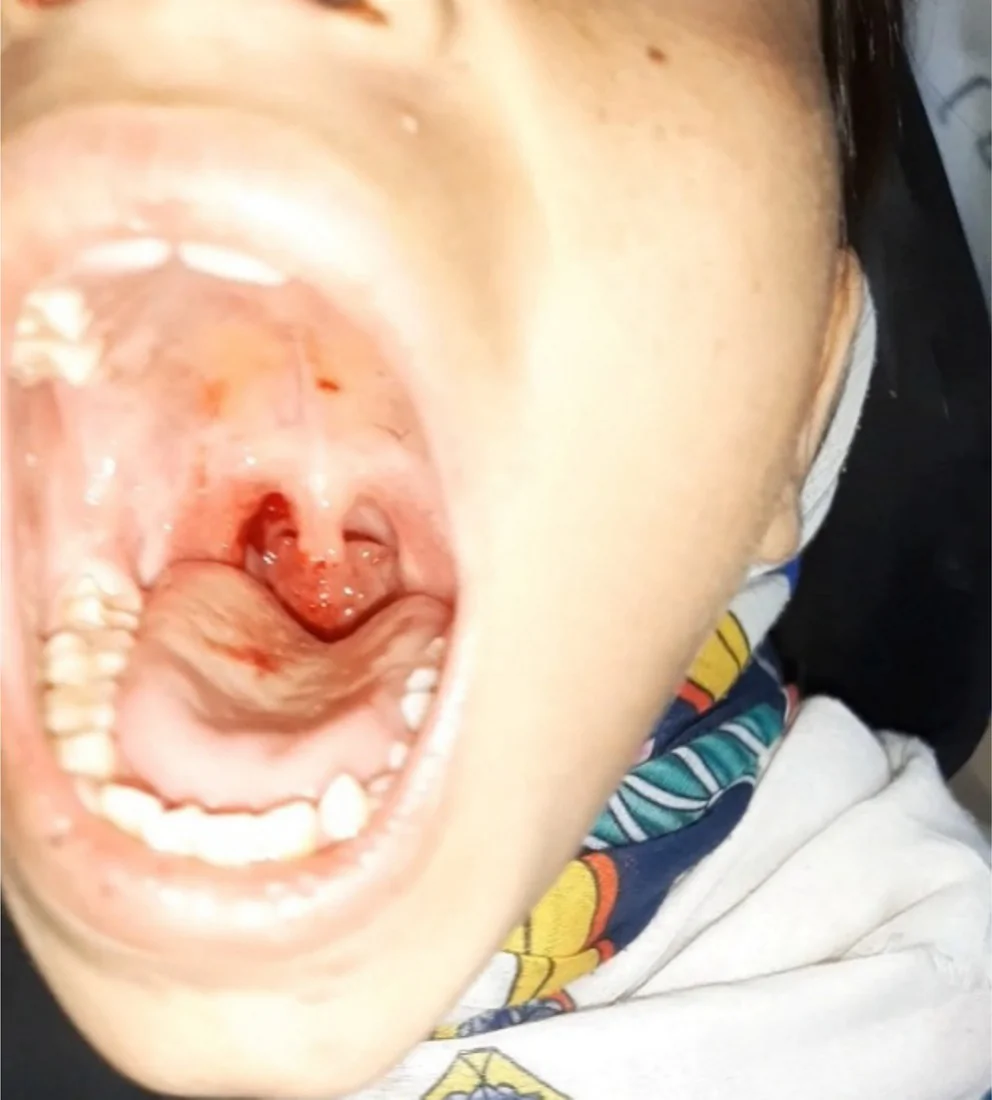
Oropharyngeal view after leech removal. Post‐removal clinical view of the oropharynx showing clearance of the parasite from the posterior pharyngeal wall, with no visible residual foreign body or active bleeding at the attachment site.

**FIGURE 3:**
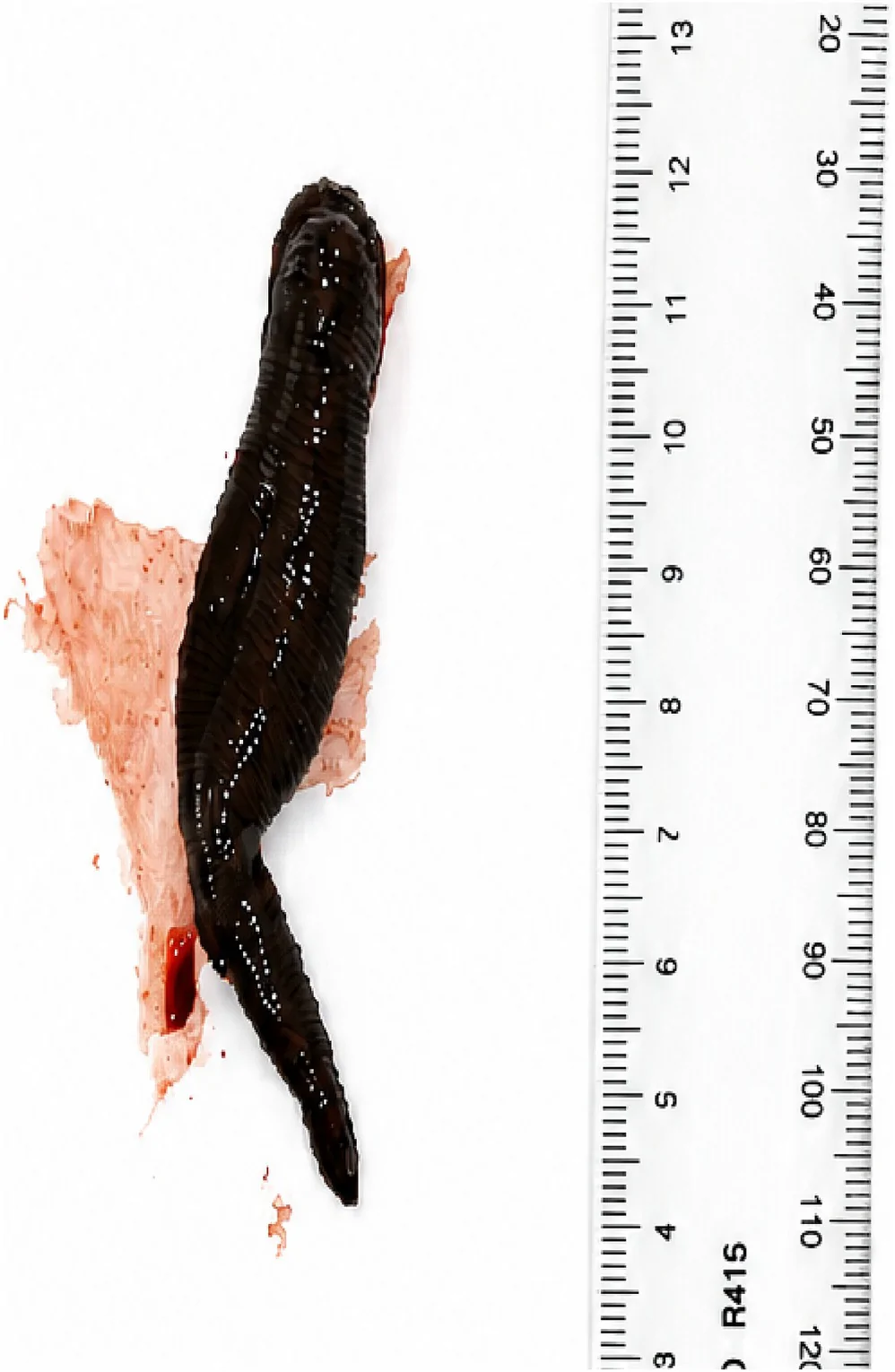
Extracted leech specimen. The live leech after removal under direct visualization using forceps. The extracted parasite measured approximately 8 cm in length.

The leech was carefully removed under direct visualization using forceps. After removal, repeat oropharyngeal examination showed clearance of the parasite, with no visible residual foreign body or active bleeding at the attachment site (Figure 2). The extracted parasite measured approximately 8 cm in length (Figure 3).

## 4 Conclusions and Results (Outcome and Follow‐Up)

Following removal of the leech, the patient experienced immediate and complete relief of dysphagia and cessation of epistaxis. Oral iron supplementation was initiated. At the 4‐week follow‐up visit, her hemoglobin level had increased from 9.7 to 12.2 g/dL. She remained fully asymptomatic, with no recurrence of nasal bleeding, swallowing difficulty, cough, foreign‐body sensation, or delayed complications. This favorable outcome emphasizes that early recognition and complete removal can rapidly resolve symptoms and prevent continued bleeding or airway complications. In children with unexplained epistaxis or dysphagia after untreated freshwater exposure, upper aerodigestive leech infestation should be considered.

## 5 Discussion

Leech infestation of the upper aerodigestive tract is an uncommon but clinically significant condition, particularly in children from rural or resource‐limited settings where exposure to untreated freshwater remains possible. Although leeches usually attach to accessible mucosal surfaces such as the nasal cavity, nasopharynx, or oropharynx, their clinical presentation can vary widely depending on the site of attachment, duration of feeding, and degree of local bleeding or obstruction. In the present case, the leech was attached to the posterior pharyngeal mucosa and extended toward the hypopharynx, producing recurrent epistaxis, progressive dysphagia, throat irritation, and cough. The history of drinking untreated stream water shortly before symptom onset was a key diagnostic clue.

Although the specimen was not preserved for morphological or molecular identification, a species of *Limnatis*—most plausibly *Limnatis paluda*—would be epidemiologically compatible with this presentation. Recent integrative taxonomic work places Afghan specimens within the *L. paluda* clade together with material from Iran, Turkey, Azerbaijan, Kazakhstan, and Uzbekistan [12]. This attribution remains presumptive; definitive identification would require examination of diagnostic morphology and/or molecular sequencing.

The clinical importance of this case lies in the unusual site of infestation and the nonspecific nature of the initial symptoms. Oropharyngeal leech infestation may be mistaken for bacterial pharyngitis or tonsillitis, a retained upper‐airway foreign body, an adherent blood clot, or peritonsillar pathology. Bacterial infection generally produces fever and mucosal erythema or exudate rather than a mobile dark lesion with persistent bleeding, whereas peritonsillar disease more often causes unilateral swelling, uvular deviation, trismus, or a muffled voice. Foreign bodies, blood clots, polyps, vascular lesions, and tumors may resemble a mucosal mass but do not move spontaneously. In our patient, the initial diagnosis was a nonspecific throat infection, and symptomatic treatment was given before the correct diagnosis was established. Similar delays have been described in previous reports, where leech infestation was recognized only after persistent bleeding, worsening anemia, respiratory symptoms, or direct visualization of a moving dark mass in the upper airway [4, 5, 6, 7, 8, 9]. The combination of untreated freshwater exposure, persistent bleeding, and a mobile attached organism is therefore diagnostically important.

The mechanism of bleeding in leech infestation is related not only to mechanical mucosal injury but also to biologically active substances secreted in leech saliva. These include anticoagulants such as hirudin, vasodilators, and local anesthetic‐like substances, which allow the leech to remain attached and feed for a prolonged period without causing severe pain. As a result, bleeding may continue even from a small attachment site and may appear disproportionate to the visible local injury. In children, prolonged feeding can lead to clinically important blood loss because of their lower circulating blood volume. In the present case, the patient had a hemoglobin level of 9.7 g/dL after removal; after oral iron supplementation, this increased to 12.2 g/dL at 4 weeks. Severe anemia requiring transfusion has been reported in both pediatric and adult patients with pharyngeal or gastrointestinal leech infestation [8, 10, 11, 13].

Dysphagia in this patient was most likely caused by direct mechanical irritation and partial obstruction of the oropharyngeal–hypopharyngeal region. The extracted leech measured approximately 8 cm, which is large enough to cause foreign‐body sensation, swallowing difficulty, cough, and gagging in a child. Although our patient had no respiratory distress, extension of a leech into the hypopharynx or larynx can potentially result in airway compromise. Previous pediatric reports have described laryngeal leech infestation presenting with stridor, dyspnea, hoarseness, and upper airway obstruction [7, 9]. Therefore, clinicians should treat suspected upper airway leech infestation as a potentially urgent condition, particularly when symptoms include respiratory distress, voice change, persistent cough, or signs of airway narrowing.

Table 1 summarizes representative pediatric cases of upper aerodigestive tract leech infestation, including their clinical presentations, treatment approaches, and outcomes. The present case is consistent with this literature; unlike several younger children who required transfusion or an airway procedure, our patient remained hemodynamically stable and recovered after direct forceps removal and oral iron supplementation.

**TABLE 1:** Representative pediatric cases of upper aerodigestive tract leech infestation.

Patient age	Country	Anatomical site	Presenting features	Treatment	Outcome	References
12 years (present case)	Afghanistan	Posterior oropharynx, extending toward hypopharynx	Recurrent epistaxis, progressive dysphagia, throat irritation, cough; Hb 9.7 g/dL	Direct forceps removal; oral iron supplementation	Complete symptomatic recovery; Hb 12.2 g/dL at 4 weeks; no recurrence or delayed complications	Current study
2 years, 10 months	Kenya	Deep oropharynx	Tiredness, mild unilateral epistaxis, one episode of hematemesis; profound anemia	Laryngoscopic removal with Magill forceps; blood transfusion and iron	Full recovery	[14]
1 year	Ethiopia	Proximal trachea, immediately below the vocal cords	Blood‐stained saliva, increasing restlessness, progressive respiratory distress	Direct laryngoscopy under general anesthesia; blunt‐forceps extraction	Full recovery; discharged on day 3	[15]
11 years	Iran	Right posterior oropharyngeal wall	Fresh blood in the mouth; 2‐week sore throat unresponsive to antibiotics	Blunt‐forceps removal after topical 10% lidocaine	No recurrent bleeding at 1 week	[16]
7 years	Uganda	Naso‐oropharyngeal junction/posterior pharyngeal wall	Epistaxis, blood‐stained saliva or hemoptysis, foreign‐body sensation, throat clearing, mild anemia	Tilley‐forceps removal without anesthesia; oral iron	Complete resolution at 2 weeks	[17]
5 years	Turkey	Pharynx	Hematemesis or vomiting of fresh blood, epistaxis, severe pallor and anemia	Urgent transfusion; removal under local anesthesia	Bleeding ceased; clinical recovery	[18]
9 years	Iran	Pharynx	Nausea, three episodes of bloody vomiting, cough	Detachment and removal after topical lidocaine	Not explicitly reported	[19]
3 years	Ethiopia	Epiglottis	Dyspnea, blood‐stained saliva, dark stool, severe anemia	Direct laryngoscopy and Magill‐forceps removal under anesthesia; whole‐blood transfusion	Respiratory symptoms resolved; discharged after 48 h	[20]
5 years	Morocco	Hard palate	Palatal swelling, blood‐stained saliva or bloody sputum	Forceps removal after local lidocaine	Asymptomatic at 1 month	[21]
8 months	India	Nasopharynx	Nasal blockage or twang, blood‐tinged discharge, mouth breathing, pallor, failure to thrive, severe anemia	Endoscopic extraction after topical lignocaine	Symptomatic, hematologic, and weight improvement at 2 months	[22]

*Note:* A dash indicates that no external reference applies to the present case.

Abbreviation: Hb, hemoglobin.

Diagnosis depends primarily on clinical suspicion and direct visualization. A careful history of recent exposure to untreated water is essential, but absence of a clear history does not exclude the diagnosis, as children may not always report water exposure accurately. Examination should include adequate visualization of the nasal cavity, oropharynx, and, when indicated, nasopharyngolaryngoscopy. The parasite may appear as a dark, mobile, smooth, or blood‐filled structure attached to mucosa, and it may be mistaken for a clot, necrotic tissue, polyp, or foreign body. In our case, the initial observation of a small dark‐colored structure on the posterior pharyngeal wall prompted more meticulous examination, which revealed the live leech.

The definitive treatment is complete removal of the leech under direct visualization while minimizing mucosal trauma and preventing fragmentation of the parasite. Forceps removal is commonly used when the leech is visible and accessible, as in the present case. For deeper or airway‐related infestations, endoscopic removal may be required, and airway preparation should be considered. Various methods, including application of hypertonic saline, lidocaine, alcohol, or other irritants, have been described to encourage detachment; however, such measures should be used cautiously in the upper airway because of the risk of aspiration, laryngospasm, or worsening airway compromise. In our patient, careful extraction with forceps resulted in immediate cessation of dysphagia and epistaxis, and follow‐up showed no recurrence of symptoms.

This case also has public health relevance. In regions where communities may rely on rivers, streams, or ponds for drinking water, leech infestation remains a preventable condition. Public awareness about avoiding untreated water, especially during outdoor activities, is important. Simple preventive measures such as boiling, filtering, or otherwise treating water before drinking may reduce the risk. Healthcare workers in rural settings should also be aware that persistent epistaxis, unexplained anemia, dysphagia, or throat foreign‐body sensation after freshwater exposure may indicate leech infestation.

The main limitation of this case is that the leech was not preserved for parasitological or molecular identification; therefore, the proposed attribution to *Limnatis paluda* remains presumptive. Follow‐up was limited to 4 weeks, although both symptomatic and hematologic recovery were documented, with no recurrent bleeding or delayed complications. Despite these limitations, the case is valuable because it documents a rare oropharyngeal presentation in a child from Herat, Afghanistan and emphasizes a diagnosis that may be overlooked in routine practice. Early recognition and prompt removal are essential to prevent continued bleeding, anemia, and possible airway complications.

## Author Contributions

**Ali Rahimi:** writing – original draft, writing – review and editing, supervision. **Said Abdul Kabir Hawari:** conceptualization, writing – original draft, writing – review and editing. **Nasar Ahmad Shayan:** writing – original draft, writing – review and editing, supervision. **Mohammad Masudi:** writing – original draft, writing – review and editing, supervision. **Seyed Abdul Ahad Wadoodi:** conceptualization, investigation, writing – original draft, writing – review and editing.

## Funding

The authors have nothing to report.

## Ethics Statement

Ethical approval for this case report was obtained from the Ethics Committee of Jami University (approval code: J.2026.1.18), Herat, Afghanistan. The study was conducted in accordance with the principles of the Declaration of Helsinki. Written informed consent was obtained from the patient's parent/legal guardian prior to inclusion, with assurances regarding confidentiality and exclusive research use of the information.

## Consent

Written informed consent was obtained from the patient's parent for publication of this case report and any accompanying images. All accompanying clinical images were reviewed and anonymized to remove identifiable facial features before submission. A copy of the written consent is available for review by the Editor‐in‐Chief of this journal upon request.

## Conflicts of Interest

The authors declare no conflicts of interest.

## Data Availability

The data supporting the findings of this case report are available from the corresponding author, Dr. Mohammad Masudi (mhmasoudy313@gmail.com), upon reasonable request.
